# Psychotherapy: A World of Meanings

**DOI:** 10.3389/fpsyg.2019.00460

**Published:** 2019-03-22

**Authors:** Cosima Locher, Sibylle Meier, Jens Gaab

**Affiliations:** Division of Clinical Psychology and Psychotherapy, Department of Psychology, University of Basel, Basel, Switzerland

**Keywords:** meaning, narrative, plausibility, psychotherapy, placebo

## Abstract

Despite a wealth of findings that psychotherapy is an effective psychological intervention, the principal mechanisms of psychotherapy change are still in debate. It has been suggested that all forms of psychotherapy provide a context which enables clients to transform the meaning of their experiences and symptoms in such a way as to help clients feel better, and function more adaptively. However, psychotherapy is not the only health care intervention that has been associated with “meaning”: the reason why placebo has effects has also been proposed to be a “meaning response.” Thus, it has been argued that the meaning of treatments has a central impact on beneficial (and by extension, negative) health-related responses. In light of the strong empirical support of a contextual understanding of psychotherapy and its effects, the aim of this conceptual analysis is to examine the role of meaning and its transformation in psychotherapy—in general—and within three different, commonly used psychotherapy modalities.

## Introduction

Psychotherapy is an effective psychological intervention for a multitude of psychological, behavioral, and somatic problems, symptoms, and disorders and thus rightfully considered as a main approach in mental and somatic health care management (Prince et al., 2007; Goldfried, 2013). But despite the wealth of empirical findings, the principal mechanisms of psychotherapy change are still in debate (Wampold and Imel, 2015). Two rival models have been contested ever since the very beginning of psychotherapy research, when some 80 years ago Saul Rosenzweig wondered, “whether the factors alleged to be operating in a given therapy are identical with the factors that actually are operating and whether the factors that actually are operating in several different therapies may not have much more in common than have the factors alleged to be operating.” (Rosenzweig, 1936, p. 412). Rosenzweig questioned the common understanding of psychotherapy, in which it is assumed that specific techniques have specific effects. This proposition was later elaborated through the work of Jerome Frank who argued that all forms of psychotherapy provide a context which enables patients to transform the meaning of their experiences and symptoms in such a way as to help them to feel better, function more favorably, and think more adaptively (Frank, 1986).

Interestingly and central to this paper, psychotherapy is not the only psychological intervention which has been associated with meaning. Following the assumption that “meaning responses are always there” (Moerman, 2006, p. 234)—i.e., in any medical and psychological treatment—the attribution of meaning has also been considered as an overarching mechanism for those treatment effects which placebo controls for in clinical trials. Thus, the attribution of a therapeutic meaning to a given intervention has a central impact on health-related responses (Barrett et al., 2006).

The contextual model of psychotherapy remains topical (Kirsch et al., 2016), and also controversial (Marcus et al., 2014). The model has been developed to propose that it is the “common factors” (e.g., client-therapist relationship, clients’ expectations, trust, understanding, and expertise) across different versions of psychotherapy that explain their effectiveness (for details, see Wampold et al. (2011)). The hypothesis for the general equivalence of various forms of psychotherapies is usually referred to as the *dodo bird conjecture* (Rosenzweig, 1936). Hence, the contextual model of psychotherapy is markedly in contrast with the long-held assumption that specific methods are at the root of psychotherapy’s effects. The assumption that psychotherapy’s effects can be reduced to incidental—or contextual—constituents, which are typically called common or unspecific factors, has been a constant in psychotherapy research (Luborsky et al., 2002; Gaab et al., 2016) but at least in terms of empirical evidence, there is sound reason and accumulating empirical support for a contextual understanding of psychotherapy (Wampold and Imel, 2015). For example, a number of meta-analyses showed that various bona fide psychotherapies, i.e., therapies with a clear treatment rationale but with very different underlying theories, aims, and methods appear to be equally effective (Spielmans et al., 2007; Cuijpers et al., 2008; Barth et al., 2013; Frost et al., 2014). In addition, opposing treatment approaches with the same treatment rationale have shown to be equally effective in a trial on clients with panic disorder (Kim et al., 2012) as much as similar treatments provided with opposing treatment rationales have shown to differ in their effects (Tondorf et al., 2017).

Building on the strong empirical support for a contextual understanding of psychotherapy (Wampold and Imel, 2015), which proposes the transformation of meaning as its central mechanisms, the aim of this conceptual analysis is to examine the role of meaning and its transformation in psychotherapy in general and in three different and commonly used psychotherapy approaches.

## In Search of a New Meaning

The main incentive to undergo a psychotherapy treatment is to change the general level of functioning, as well as to reduce the symptoms of suffering (Strong and Matross, 1973). Clients’ belief that they are unable or incapable of solving disturbing problems contributes to demoralization and feelings of confusion, despair, and incompetence (Vissers et al., 2010) or as Frank (1986) put it: “Often an important feature of demoralization is a sense of confusion resulting from the client’s inability to make sense out of his experiences or to control them, leading to the commonly expressed fear of going insane” (p. 341). This demoralization is not only a shared aspect of various psychological disorders, but can also be considered as a starting point for change in psychotherapy. Therapeutic change is thereby accompanied by clients “working through” their problems, gaining insight, achieving personal fulfillment, and becoming self-actualized, eventually transforming their problems and symptoms, self-perception, and experiences with their social environment (Evans, 2013; Krause et al., 2015).

Frank (1986) stated that psychotherapy seeks to help clients to transform the meanings of their problems and symptoms and to overcome confusion with newly acquired clarity, i.e., by offering a narrative that links symptoms with hypothesized causes and providing a collaborative procedure for overcoming the suffering. Likewise, Wampold (2007) defined the core of psychotherapy in the transformation of non-adaptive explanations for their problems into new and more adaptive ones. Also, Dan Moerman (Moerman, 2002) stated that “it sounds reasonable to me to say that psychotherapy evokes meaning responses” (p. 94) and that psychotherapy supports clients to create their stories and myths, although therapists are not considered a mandatory requirement for this (Moerman, 2002). It should be noted that other mechanisms underlying positive response to psychotherapy have been proposed, e.g., reward mechanisms in psychotherapy (Northoff and Boeker, 2006; Panksepp and Solms, 2012).

Considering processes of change in diverse interventions, narratives are thought to be created in order to render the demoralization less painful and promote remoralization (Moerman, 2002). In this perspective, the therapists help their clients to give new meanings to their experiences or stories they tell, the language they use, and the beliefs they have (Shaw, 2010).

Similar processes have been proposed to underlie placebo responses, which are “most likely to occur when the meaning of the illness experience is altered in a positive direction” (Brody, 2000). These beneficial changes in meaning occur when three core conditions are present, which again resemble those proposed in the context of psychotherapy: (1) the clients feel listened to and receive a satisfactory, coherent explanation of their mental suffering and demoralization; (2) the client feels care and concern from the therapist; and (3) the clients feel an enhanced sense of mastery and control over their mental suffering (i.e., remoralization). A direct implementation can be seen in so-called narrative therapies, which are defined as “an approach that focuses on client stories with the goal of challenging existing meaning systems and creating more functional ones” (Kropf and Tandy, 1998). Narrative approaches have come to a central role in systemic family therapy (Carr, 1998; Wallis et al., 2011), emphasizing the role of language and how it affects the way clients frame their ideas of self and identity, while the therapist directly deals with clients’ concerns and the meaning of the worlds they live in (Besley, 2002). Furthermore, it has been assumed that relying on the clients’ individual narratives is more significant than focusing on a pathological psychiatric diagnosis (Gysin-Maillart et al., 2016). Of course, narrative therapies should not be mistaken as the exception of (dodo) rule, i.e., to be instances of “specific” therapies, but rather as possibilities to operationalize the rule in real life, i.e., to employ meaning processes in psychotherapy. Accordingly, it has been shown that meaning-making through language enhances clients’ well-being after a traumatic experience—which mainly stems from the connection, abstraction, and reflection of the whole experience (Freda and Martino, 2015; Park et al., 2016).

## Co-Construction of Narratives

To bring about these meaning transformations, psychotherapists mostly rely “on the use of words to form attitudes or induce actions” (Frank, 1986). The overarching definition of a narrative is as a sequence of actions and events that involves a certain number of human beings as characters or actors (Bruner, 1990). However, narratives differ from conventional discourse forms in variety of ways as narratives have an inherent sequentiality which is more than a just chronological sequence of lived experiences as it links the past to the present and future (Bruner, 1990). A narrative creates a coherent whole out of a sequence of events (Mattingly, 1994). In order to report an experience in a meaningful way, the protagonists not only focus on overt characteristics, but also reflect on their beliefs and feelings and how these are connected to their personal life in general (Ochs and Capps, 1996). In regard to a broader social context, Justman (2011) argues that any information about a given intervention has an influence on the experience of clients receiving the intervention, which of course is particularly applicable for any form of psychotherapy.

In this understanding, any psychotherapy alleviates the symptoms of a target illness through meaning transformation. Thus, the relationship between narrative and illness—understood as the subjective experience of a given pathological process and their embedment in social context (Engel, 1977)—should briefly be exemplified. An illness narrative is defined as the important channel through which the meaning of an illness is created (Kleinman, 1988). Thus, illness narratives do not merely reflect an illness experience, but they have been shown to be clinically relevant as they significantly impact on health behaviors and coping strategies as well as treatment outcomes (Broadbent et al., 2004; Horne et al., 2007; Frenkel, 2008; Galli et al., 2010).

Furthermore, narratives are interdependent with the social context, so that therapists should use “images from the same sensory modality as that of the patient’s own imagery” (Frank, 1986). The alternative narratives which are provided to the client in order to offer new and adaptive perspectives should be different but not too far off the client’s general beliefs (Wampold, 2007) as much as exploring the clients’ narrative through attentive listening—both considered as key factors of healing processes (Egnew, 2005; Mauksch et al., 2008)—as they allow the therapist to reformulate and interpret the perceived meanings in a way that the clients can connect them with their personal conception of the world on the background of their beliefs and culture (Strupp, 1986). Considering narratives in the psychotherapeutic encounter, the importance of the therapist’s and client’s co-construction of the narrative has been considered as a significant element of psychotherapy (Brody, 1994) as jointly developed narratives significantly contribute to new forms of self-understanding and of being in the world (Levitt et al., 2016). According to Brody (1994), a shorter form of the client’s possible plea to the psychotherapist might be, “My story is broken; can you help me fix it?” (p. 85). Recovery in this understanding includes a deepening of a client’s experience and the development of a more comprehensive and coherent personal narrative (Lysaker et al., 2011).

## Truth Matters?

As outlined, the transformation of non-adaptive narratives into more adaptive ones is central to the contextual understanding of psychotherapy. This raises the question of the relationship between the narratives, i.e., their quality to induce subjective understanding, and the “real world,” i.e., the actual facts of a client’s life and actual causes of symptoms (Kendler et al., 2011). Importantly, two kinds of narratives should be distinguished. First is the narrative behind the therapeutic approach, i.e., the healing narrative. Frank reasoned “that the chief criterion of the truth of any psychotherapeutic formulation is its plausibility” (Frank, 1986). Hence, the explanation for why the treatment works should be plausible for both the therapist and the client. When the healing narrative is credible for the clients, they will discern and pick up on the aims and goals of therapy. Common factors associated with the healing narrative are for example the provision of an explanation for the client’s problems, therapeutic actions that are consistent with the explanation, as well as education (Kirsch et al., 2016).

A second narrative is the client narrative that may emerge from therapy. This kind of narrative amounts to the actual change in the personal story, i.e., explanations that clients in therapy come to acquire about their own personality and reasons for their suffering. Common factors related with the client narrative are insight, corrective emotional experience, emotion regulation, and mindfulness among others (Kirsch et al., 2016). Forming personal experiences into a narrative has further been associated with both physical and mental well-being and, accordingly, “psychotherapy is a more formal venue that often involves putting together a story” (Pennebaker and Seagal, 1999). While this concept resonates with the now sadly predominant concept of “truthiness” (i.e., the quality of stating concepts or facts one wishes or believes to be true, rather than concepts or facts known to be true) in everyday life (Metcalf, 2005), the understanding of plausibility in the context of a narrative is basically subjunctive or put otherwise: something is subjectively perceived to be possible (Kleinman, 1988; Bruner, 1990). Further, this subjunctivity emphasizes that the anticipated future course is indispensably reported with some level of uncertainty, thus “to make a story good, it would seem, you must make it somewhat uncertain, somehow open to variant readings, rather subject to the vagaries of intentional states, undermined” (Bruner, 1990). Likewise, Frank (1986) pointed out that “life histories do not provide adequate causal explanations of clients’ symptoms” (p. 343) and Jopling notes that “insights such as these may strike clients as entirely plausible and coherent, but neither plausibility nor coherence are, in themselves, a guarantee that the insights are true and that they fit the facts” (Jopling, 2011). This corresponds with the idea that a client narrative emphasizes possibilities rather than predefined certainties (Bruner, 2004) and that it not only copies reality as it is, but gives meaning to it through language (Bruner, 1990). In turn, constructing the reality according to own beliefs and experiences affects as well as constitutes one’s self-perception. Accordingly, a client narrative is not only considered a personal report, but it also creates the identity of the story-teller (Ricoeur, 1991). The self is reformed, which means that narrative and self are actually inseparable (Ochs and Capps, 1996).

With these considerations in mind, the necessity of truth of an adaptive explanation for the client’s healing process comes into question. In this regard, it has been assumed that it might not be the truth itself that makes a narrative meaningful, yet rather its plausibility and “the extent to which a client is convinced by it” (Frank, 1986). A plausible narrative for a mental disorder or a therapeutic change, respectively, invokes new information and is related to previous explanatory structures and networks of a client, when the new explanation is not too divergent from the previous one and takes a client’s perception of the world into account (Wampold, 2007). Likewise, for clients who accept the treatment rationale, psychotherapeutic success occurs more quickly and psychotherapy outcomes are significantly better than for those who do not agree with the treatment rationale (Addis and Jacobson, 2000; Overholser et al., 2010). However, the position that truth of a narrative is not the prerequisite for its meaningfulness does of course and in no way preclude psychotherapy and psychotherapists from the ethical obligations to respect clients’ autonomy. First, clients should not be deceived by providing false, but plausible narratives under any circumstances. Second, therapists should be aware to not withhold proven but possible implausible evidence about psychotherapy and psychotherapy change (Blease et al., 2016; Gaab et al., 2016; Trachsel and Gaab, 2016).

## Meaning Transformation in Psychotherapeutic Schools

Each psychotherapeutic school relies on a specific treatment theory, which addresses the connection between symptoms and hypothesized causes, as well as the process of therapeutic change. This treatment theory defines which treatment constituents are to be considered characteristic and which incidental (Grünbaum, 1981). Although the various therapy approaches not only differ substantially in their operationalization of their constituents, but also in assignment to be either characteristic or incidental (e.g., the therapeutic alliance, Flückiger et al. (2012)), they explicitly or implicitly promote a meaningful transformation regarding how clients understand and cope with their problems and symptoms, which in turn affects their self-perception and the interaction with their social environment. In the following, this shall be exemplified on three psychotherapeutic approaches. We decided to focus on three prominent psychotherapeutic approaches, with no claim to be complete in terms of therapeutic theories and methods. The following arguments guided our decision: first, the chosen psychotherapy approaches differ substantially in their underlying treatment theory; second, we decided to not focus on the link between psychodynamic psychotherapy with the “narrative feature of psychotherapy ‘which may be’ its main therapeutic engine” (cited from Blease, 2015, p. 178) since this has been discussed elsewhere (Jopling, 2011; Blease, 2015); third, popular third wave approaches (e.g., dialectical behavior therapy or acceptance and commitment therapy) conceptualize cognitions and cognitive thought processes as a way of “private behavior” (Hayes et al., 2006), focusing primarily on the function of cognitions (Churchill et al., 2010). We assume that the reflections on cognitive therapies will exhibit at least some comparable inferences.

## Cognitive Therapies

The cognitive approach is based on the assumptions that the cognitive representation of clients’ experiences influences how they respond, act, and feel and that humans have the potential to metacognitize, thus to observe and change their thoughts and beliefs through reflection and practice, resulting in a different perception of one’s symptoms, self, and social environment (Beck, 1996). The process of transformation through metacognition is embedded in a caring, collaborative, and respectful therapeutic relationship (Alford et al., 1998; DeRubeis et al., 2001; Dobson and Dozois, 2001; Beck, 2005) and therapists should be competent not only in technical but also in interpersonal skills (Beck and Padesky, 1989; Gaston et al., 1998).

The cognitive approach was initially formulated as a treatment for depression; later, it became very popular as an approach for treating a multitude of other mental disorders including anxiety disorders and posttraumatic stress disorders. However, even from its early days, reception to cognitive therapy included the criticisms that the approach faced “formidable conceptual, methodological, and empirical difficulties” (Coyne and Gotlib, 1983) and that “it has the force of a good story, and does not ask us to believe in any cognitive mechanism beyond those that have been familiar to playwrights and novelists for centuries.” (Lang, 1988).

Interestingly, the conceptualization of clients’ cognitions soon developed from being a merely covert behavior or the result of erroneous information-processing to the notion that clients are constructors of their own representation of the world and that the reality is “a product of personal meanings that individuals create” (Meichenbaum, 1993). As such the cognitive therapist “helps clients to construct narratives that fit their particular present circumstances, that are coherent, and that are adequate in capturing, and explaining their difficulties” (Meichenbaum, 1993). Besides this kind of meaning, where the cognitive therapist and the client create meaningful narratives, there is also another kind: the meaningfulness or plausibility of the therapy itself. In an earlier publication, Don Meichenbaum—a major proponent of cognitive therapies—also addressed the supremacy of plausibility over validity stating that “although the theory (i.e., Schachter’s model of emotional arousal) and research upon which it is based have been criticized (…), the theory has an aura of plausibility that the clients tend to accept. The logic of the treatment plan is clear to clients in light of this conceptualization” (Wampold and Imel, 2015).

Addressing the lack of differences in efficacy between cognitive and clearly non-cognitive treatments for panic disorder and the lack of a clear confirmation of the validity of underlying cognitive theories, Roth (2010) noted that “there is little doubt that therapists have been able to greatly help clients in spite of giving rationales that have turned out to be questionable or demonstrably false” (Roth, 2010). In the same vein, it is interesting to note that although the hyperventilation theory (i.e., clients are instructed to counteract hyperventilation by breathing slowly and abdominally, which is expected to increase Pco₂) has been falsified as well as the suffocation false alarm theory (i.e., clients are thought to lower their Pco₂) is difficult to falsify (Roth et al., 2005), treatments on the basis of these—interestingly opposing!—theories have been shown to be equally effective in the treatment of panic disorder (Kim et al., 2012). As a solution of the ethical conundrum to provide effective therapies despite them being false or questionable, Roth (2010) referred to Williams James’ pragmatic approach (James, 1896) and to “simply teach clients a practice that has prevented attacks in others (such as breathing control) but without its pseudoscientific rationale, asking clients to test whether that practice helps them as individuals.”

## Systemic Therapy

Systemic therapy is based on the assumption that a system is constructed by shared representations of realities, building a consensus on how to interpret the internal and external environment (Reiss and Olivieri, 1980) and that this collective perception is largely determined by the emotional experiences of the members of the given system. The pattern of meaning within a system is mediated by its use of language and its narrative tradition. Behaviors, symptoms, and expression of emotions are thus not considered as objective and independent entities, but rather as functional to the mutual relationships within a system and as constructed through the actions and communications of and between its members. Therefore, systemic therapy intends to change the shared patterns of meaning and definitions of realities in the context of the particular system, i.e., therapists aim to understand and accept the individual pattern of meaning as much as the systems’ narrative about their reality. Neutrality and unconditional therapeutic curiosity (Cecchin, 1987), i.e., the therapist does not side or support individual members of a given system, are thought to encourage all involved members of a system to share ideas and problem perceptions, while subjective truths are appreciated in the same way. This attitude of neutrality is contrary to the idea that a system could be understood entirely, i.e., that “truth” exists, and thus attempts to explore the systems’ narratives (Selvini et al., 1980).

The meaning-changing nature of systemic therapy shall be exemplified on the basis of a commonly used method in systematic therapy, the “genogram”, which consists of a structural diagram of a family’s generational relationship system using specific symbols for illustration (Guerin and Pendagast, 1976). The “genogram” aims to unravel idiosyncratic perceptions, trigger the unfolding of shared narratives with a given system, and capture the communicative meaning of behavior, symptoms, or expression of emotions (McGoldrick and Gerson, 1985). When relationship patterns become apparent and members of a given system are challenged to perceive reality by another perspective, this can result in new illness narratives and eventually new meanings (Satir et al., 1991). Another approach in systemic therapy to change meaning is to reframe communication without changing its content (Watzlawick et al., 2011). For example, the otherwise negatively and non-adaptive connoted behavior of acting-out is reframed as functional to make yourself heard in the context of a demanding and bullying school environment, thus transforming a formerly non-adaptive meaning of symptom into a new and more adaptive justification. Also, systemic therapy makes use of externalizing, i.e., to differentiate a problem and the identity of the client in order to enable a new context of meaning and to change the assumptions of what is driving and maintaining the problem (White et al., 1990). According to De Shazer’ (1985) solution-focused approach, a system is viewed as having all resources that it needs for solving the problem but it is not using them currently. Thus, idiosyncratic meanings of a problem on the one hand are thought to underlie the presenting problems and on the other hand are also considered as the starting point for change: building new shared narratives involve all relevant members of the system and activate individual processes.

## Person-Centered Therapy

The subjective experience of a person, i.e., the self-concept, is both the starting point as much as the therapeutic focus of the person-centered approach which is based on the principles of humanistic psychology. The person-centered approach originated in the works of Carl Rogers, who defined the necessary and sufficient condition for personality change (Rogers, 1957). The self-concept is viewed as fluid and associated with changing idiosyncratic interpretations and attributions of subjective meanings. The driving force behind any change is the self-actualizing tendency for development, enhancement, and growth (Rogers and Carmichael, 1951). Accordingly, a discrepancy between self-concept and the actual experience leads to incongruence, i.e., a state of tension and internal confusion, resembling Frank’s term demoralization (Frank, 1986). This incongruence can either be the starting point for personality change and development or—in case of too large a discrepancy—be distorted, i.e., denied, biased, and not fully represented in experience.

The aim of person-centered therapy is congruence, i.e., to enable the clients to understand their own experiences and to be able to integrate them with their self-concept (Rogers and Carmichael, 1951). A meaning transformation is understood as the clients revising their self-concept in a way to allow congruence with their experience (Rogers, 1957). In this therapeutic process, the therapist is central and thus acknowledges the subjective experience of the client as much as specific techniques are only advocated when they “become a channel for communicating the essential conditions” (i.e., empathy, congruence and unconditional positive regard) (cited from Rogers, 1957, p. 247). Thus, in person-centered therapy, the therapist and client construct a shared narrative as the therapist empathically understands the client’s inner representation of its experience and to carefully offer meanings to the client’s experience of which the client is scarcely aware (Rogers, 1957).

## Conclusion

Based on the contextual understanding of psychotherapy, we set out to examine the role and construction of meaning as a means to induce change in general and in three different psychotherapy approaches. The described psychotherapeutic approaches differ in their etiological assumptions and their therapeutic implications, but clearly all share the aim to promote a meaningful transformation in order to generate convincing narratives that “persuasively influence clients to accept more adaptive explanations for their disorders and take ameliorative actions” (Wampold, 2007).

However, the exemplified approaches differ with regard to the extent this is communicated in both the treatment rationale and to the clients. Therefore, different kinds of psychotherapy can be distinguished by the way in which they *explicitly* engage and lead to transform clients’ meaning in more adaptive ways. Considering the importance of the transformation of meaning through narratives in psychotherapy and the varying degree this is openly defined as a characteristic constituent of the given approach and communicated to clients, we believe that psychotherapy would benefit from acknowledging this within education and training, and (arguably) in communicating this to clients.

The described therapeutic schools are all placed in an interpersonal context marked by empathy, warmth, cooperation, and transparency (Langhoff et al., 2008). However, different emphases of the therapist’s and client’s roles are apparent. In cognitive therapies, the therapists assist their clients in constructing narratives that fit their perception of the world and their particular present challenges (Meichenbaum, 1993). In systemic therapy, therapists aim to understand and accept how each member of a system understands reality and which unique narratives describe the current problem (Selvini et al., 1980), while person-centered therapists promote a shared and empathetic understanding of clients’ narratives (Rogers, 1957).

The current conceptual analysis has the limitation to focus only on a selective choice of therapeutic approaches, with no claim to be complete regarding therapeutic theories, rationales, techniques, and strategies (Blease, 2015). However, the chosen psychotherapy approaches are illustrative as they differ significantly in focus, underlying treatment theory and paradigmatic orientation.

To conclude, the meaning and their underlying narratives matter—regardless of the specific psychotherapy approach. However, this importance is not equally well acknowledged by the examined approaches or to rephrase this observation in the terms of Grünbaum’s (Grünbaum, 1981; Howick, 2017) definition of intervention constituents and with regard to Rosenzweig’s (1936) early and seminar observation: The characteristic factors that actually are operating in several different therapies—the transformation of meaning—may not have much more in common than have the factors alleged to be operating. The ethical obligation at hand is to make these characteristic elements of psychotherapy, which promote the change from non-adaptive into adaptive explanations, allowing the client to feel better, function more favorably, and think more adaptively, transparent in both, therapeutic manuals and the informed consent of clients (Blease et al., 2016; Gaab et al., 2016; Trachsel and Gaab, 2016).

## Author Contributions

CL, SM, and JG conceived and designed the conceptual analysis. CL drafted the paper. CL, SM, and JG wrote the final paper, critically revised the manuscript and gave important intellectual contribution to it.

### Conflict of Interest Statement

The authors declare that the research was conducted in the absence of any commercial or financial relationships that could be construed as a potential conflict of interest.
